# Laboratory-acquired Scrub Typhus and Murine Typhus Infections: The Argument for a Risk-based Approach to Biosafety Requirements for *Orientia tsutsugamushi* and *Rickettsia typhi* Laboratory Activities

**DOI:** 10.1093/cid/ciy675

**Published:** 2018-08-10

**Authors:** Stuart D Blacksell, Matthew T Robinson, Paul N Newton, Nicholas P J Day

**Affiliations:** 1 Mahidol-Oxford Tropical Medicine Research Unit, Faculty of Tropical Medicine, Mahidol University, Bangkok, Thailand; 2 Lao-Oxford-Mahosot Hospital–Wellcome Trust Research Unit, Mahosot Hospital, Vientiane, Lao People’s Democratic Republic; 3 Centre for Tropical Medicine and Global Health, Nuffield Department of Clinical Medicine, Churchill Hospital, Oxford, United Kingdom

**Keywords:** scrub typhus, murine typhus, laboratory-acquired infections, biosafety

## Abstract

This study examined the literature on laboratory-acquired infections (LAIs) associated with scrub typhus (*Orientia tsutsugamushi*) and murine typhus (*Rickettsia typhi*) research to provide an evidence base for biosafety and biocontainment. Scrub typhus LAIs were documented in 25 individuals, from 1931 to 2000 with 8 (32%) deaths during the preantibiotic era. There were 35 murine typhus LAI reports and no deaths. Results indicated that the highest-risk activities were working with infectious laboratory animals involving significant aerosol exposures, accidental self-inoculation, or bite-related infections. A risk-based biosafety approach for in vitro and in vivo culture of *O. tsutsugamushi* and *R. typhi* would require that only high-risk activities (animal work or large culture volumes) be performed in high-containment biosafety level (BSL) 3 laboratories. We argue that relatively low-risk activities including inoculation of cell cultures or the early stages of in vitro growth using low volumes/low concentrations of infectious materials can be performed safely in BSL-2 laboratories within a biological safety cabinet.

## Teaser

The highest-risk activities for scrub/murine typhus laboratory-acquired infections were working with infectious laboratory animals. Eight scrub typhus deaths occurred during the preantibiotic era. Risk-based biosafety approaches would improve efficiencies of in vitro/in vivo growth of scrub/murine typhus.

Scrub typhus is caused by *Orientia tsutsugamushi* (previously known as *Rickettsia orientalis* and *Rickettsia tsutsugamushi*) and is a member of the scrub typhus group antigenic complex along with the related *Candidatus Orientia chuto* [1, 2]. Murine typhus or endemic typhus is caused by *Rickettsia typhi* (previously known as *Rickettsia mooseri*) and is a member of the typhus group antigenic complex along with *Rickettsia prowazekii*, the causative agent of epidemic typhus. Scrub typhus is a common cause of acute febrile illness in the Asia-Pacific region [2–4] but has recently been described outside of the so-called “tsutsugamushi triangle” [5, 6]. Murine typhus has a worldwide distribution with significant burden of disease in the Americas, Asia, and Australasia [2, 4, 7, 8]. Both diseases remain foci for laboratory investigations to this day due to their importance as causes of acute febrile illness.

*Orientia* spp and *R. typhi* are gram-negative, obligate, intracellular bacteria that grow in the cytoplasm or vacuoles of host cells and divide by transverse binary fission [3]. In vitro propagation of these organisms necessitates growth in cell cultures [9] or in vivo propagation in laboratory animals [10–14]. There are 3 main prototype strains of *O. tsutsugamushi—*Gilliam, Karp, and Kato. The Karp strain was isolated in New Guinea in 1943 [15], the Gilliam strain in 1944 in Burma (present-day Myanmar) [16], and the Kato strain in 1952 in Japan [17]. The Gilliam and Karp strains were among several strains cultured from military personnel patients with scrub typhus in the Asian and Pacific theaters during World War II when it was a major cause of illness in Allied and Japanese troops [18, 19]. *Rickettsia typhi* was first described as murine typhus in 1926 by Kenneth Maxcy, by isolating the organism from the blood of patients and by comparing their antigenic characteristics to those of *R. prowazekii* and *Rickettsia rickettsii* (Rocky Mountain spotted fever) [20].

As with all human pathogens, culture of *Orientia* spp or *Rickettsia* spp in vitro or in vivo carries the intrinsic hazard of infection of laboratory and ancillary staff (known as laboratory-acquired infections [LAIs]) via parental inoculation by accidental self-inoculation or needle-stick incident, animal bite, or inhalation of infectious aerosols generated during laboratory procedures or incidents [21–24]. Importantly, *Orientia* spp and *Rickettsia* spp are susceptible to antibiotic therapy with doxycycline as first-line treatment, and azithromycin as an alternative treatment such as in the case of infections during pregnancy [25, 26] although there have been treatment failures with azithromycin in murine typhus (Newton et al [27]).

This study examined LAIs associated with scrub typhus and murine typhus research including the history, current practice, and regulatory requirements to provide an evidence base on matters of biosafety and biosecurity. Furthermore, we suggest risk-based safe and sustainable in vitro culture procedures that are suitable for low-resource settings where the diseases are endemic.

## METHODS

The review process used published information obtained via PubMed and personal anecdotes. The search was conducted on articles cited in PubMed up to 10 September 2017 combining the search terms “scrub typhus,” “typhus,” “murine typhus,” and “laboratory-acquired-infection” or “accidental infection.” The titles and abstracts were screened and the full texts of relevant articles were reviewed. Manual screening of the reference list of relevant articles was also performed. One author (S. D. B.) reviewed abstracts and titles from all search results to assess eligibility. The full article was obtained and then all authors conferred on matters of eligibility that included information regarding the circumstances of the LAI including the infecting organism, year, who was infected, and location.

## RESULTS

### Scrub Typhus LAIs

Scrub typhus LAIs were documented in 25 individuals, identified in 11 reports from the period 1931–2000 (Tables 1 and 2). In these reports, there were 8 (32%) deaths and these were all in the preantibiotic era (pre-1950s). The primary routes of infection were cutaneous (accidental self-inoculation and animal bites, 24%) and aerosols (16%), but another 60% were unstated. All LAI reports were in laboratory and ancillary staff as a result of laboratory incidents or procedural errors, with no scrub typhus infections reported in individuals working near but outside of a laboratory.

**Table 1.** Summary of the Features of *Orientia tsutsugamushi* and *Rickettsia typhi* Laboratory-acquired Infections

Infection	Total Reports	Total Infections	Outcome		Cause		
			Recovered	Died	Aerosol	Cutaneous	Not Stated
Scrub typhus	11	25	17 (68%)	8 (32%)	4 (16%)	6 (24%)	15 (60%)
Murine typhus	10	35	35 (100%)	0	34 (97%)	1 (3%)	0

**Table 2.** Summary of Published *Orientia tsutsugamushi* Laboratory-acquired Infections

Year	Location	Route	No. Infected	Outcome	Patient Details	Time to Illness Onset or Death	Circumstances	Reference
1931	Japan	Self-inoculation	2	Fatal	2 laboratory technicians	NA	Slipped while inoculating a rabbit with *Rickettsia orientalis*.	[28] (cited by [29])
1940	Australia	Not stated	1	Nonfatal	Animal technician	NA	Assistant at W. G. Heaslip’s laboratory in South Australia where *O. tsutsugamushi* vector experiments were performed.	[30, 31]
1943	Australia	Self-inoculation	1	Fatal	Female, 32 y	23 d	Walter and Eliza Hall Institute, Melbourne. Died 20 May 1943. Self- inoculation of *O. tsutsugamushi* when inoculating a mouse.	[30, 32]
1943–1947	India/US	Self-inoculations; wounds; rat bite; contamination of conjunctiva; aerosol; possible mite bites	11	Nonfatal	Various	NA	No specific details of the nonfatal cases, only the location of the infections: • US Typhus Commission in Assam, India (1943) • Members of British Scrub Typhus team at Imphal, India (1945) • Technicians at the National Institute of Health at the Rocky Mountain Laboratory (1944) • Naval Medical Research Institute, Bethesda, Maryland (1945) • Army Medical School, Washington, DC (1947)	[30]
1944	US	Laboratory accident		Fatal	Male	NA	Rocky Mountain Laboratory, Hamilton, Montana. Died 28 September 1944.	[30]
1945	US	Laboratory accident		Fatal	Male	NA	Lederle Laboratories, Pearl River, New York. Died 22 October 1945.	[30]
1944	US	Laboratory accident		Fatal	Male, 30 y	NA	National Institutes of Health, Washington, DC. Died 20 October 1944.	[29, 30]
1945	US	Necropsy	1	Fatal	Male, pathologist		14th Evacuation Hospital, Assam, India, died in US on 16 August 1945. Performed a necropsy on a scrub typhus patient who died of scrub typhus at a hospital near Ledo a few days earlier.	[29, 30]
1945	UK	Animal bite	1	Nonfatal	Female, 42 y	14 d	Bitten by cotton rat on finger during intranasal inoculation of *O. tsutsugamushi* due to anesthetic failure.	[33]
1945	UK	Aerosol	1	Nonfatal	Male, 28 y	NA	Bubbles formed on Petri dishes containing *O. tsutsugamushi* necropsy materials for vaccine seed productions causing splash of droplets, causing infectious aerosols.	[33]
c. 1945	UK	Self-inoculation	1	Nonfatal	Female, 28 y	11 d	Accidental self-inoculation to finger with a broken Pasteur pipette used for intranasal inoculation of rats with *R. orientalis*.	[33]
1945	UK	Aerosol	1	Nonfatal	Female, 28 y	? 15 d	Washing of Petri dishes that had contained *O. tsutsugamushi*– infected rat lung homogenates that had not been previously autoclaved, resulting in aerosol exposure.	[33]
1947	US	Aerosol	1	Fatal	Male, 31 y	NA	Working with yolk sac material of *R. orientalis*, resulting in aerosol exposure.	[34]
c. 1996	Korea	Self-inoculation	1	Nonfatal	Female, 22 y	7 d	Accidental self-inoculation with *O. tsutsugamushi* while treating confirmed scrub typhus patient.	[35]
c. 2000	Korea	Aerosol	1	Nonfatal	Male, 23 y	12 d	Homogenization or ultrasonication procedure with *O. tsutsugamushi* resulting in aerosol exposure.	[36]

Abbreviations: NA, not available; UK, United Kingdom; US, United States.

Laboratory-acquired infections with these organisms were often the result of accidental self-inoculation or skin puncture [28,32,33,35,37], animal bite [33], or aerosol exposure to high concentrations of the organisms in animal tissues collected at necropsy for vaccine production [33, 37–39], and all occurred in research facilities. The earliest recorded LAI was in 1931 when a technician in Japan sustained an accidental self-inoculation injury while inoculating a rabbit with *R. orientalis* [28], cited by Pike [29] (Table 2). Not surprisingly, the majority of LAIs occurred in the 1940s when there was a significant imperative to develop a scrub typhus vaccine to reduce Allied troop infections in the Asian theater of the Second World War. From the 1950s onward, there were only 2 reported LAIs involving scrub typhus, with no recorded fatalities. The last reported scrub typhus LAI was in 2001 and was caused by an aerosol exposure following ultrasonication without suitable aerosol control measures [36].

In 1946, Van den Ende and colleagues [33] detailed a series of 4 scrub typhus LAIs that occurred at the National Institute for Medical Research in the United Kingdom (NIMR) during a period of intense vaccine production research in the early 1940s. All staff recovered from their infections and full case details are reported. The report detailed what they described as “elaborate precautions” to prevent staff infections as well as all staff members wearing gowns, rubber gloves, dust respirators, and eye shields [33]. The majority of these LAIs were attributed to infected animal bites, self-inoculation in the process of animal infection, inadequate decontamination procedures, and/or exposure to infectious aerosols during animal necropsy procedures [33].

Fatalities have been attributed to scrub typhus LAIs. A prominent scrub typhus LAI fatality was that of Dora Lush, an Australian microbiologist working on the development of scrub typhus vaccine at the Walter and Elisa Hall Institute in Melbourne under the instruction of the Nobel Laureate Sir Frank MacFarlane Burnett [32]. In April 1943, Lush sustained a self-inoculation injury to the finger with a needle containing *O. tsutsugamushi* Karp strain while inoculating a mouse, and died 4 weeks later [40]. It was noted in her obituary that she was one of several staff infected with murine typhus while working at the NIMR, and noted that the infection “confirms only too well the lack of cross-immunity between typhus and scrub typhus” [40]. Philip [30] documented deaths following *O. tsutsugamushi* LAI (Table 2); however, the details of the LAI circumstances were not provided.

Philip [30] noted that nonfatal infections occurred in personnel in other laboratories located in Australia, Asia, and the United States during the Second World War. One well-described case occurred at Dr W. G. Heaslip’s laboratory near Adelaide, Australia, in February 1940, where investigations into scrub typhus infections in Queensland [31] and the role of different species of trombiculid mites as the scrub typhus in Queensland and New Guinea were performed [31, 41]. Heaslip provided a detailed account of the case, stating that “a laboratory assistant, whose duties included the inoculation of mice with the blood of the fever cases and the subsequent examination of these animals, contracted the disease, he had 11 days of fever, a rash appeared on the 5th day and a sore was noted on his finger, with lymphangitis extending up to the elbow. Mice were inoculated with his blood and those inoculated during the febrile period became infected and died with the typical post mortem appearances; also his blood serum agglutinated *Proteus OXK* in high dilutions” [31]. Other nonfatal infections were reported by Philip [30]: The causes of these nonfatal cases were accidental self-inoculation into hands in an attempted catch of falling syringes, wound in finger through a rubber glove by the broken end of a contaminated pipette, rat bite on finger, contamination of abrasions on fingers, and possible contamination of conjunctivae of eyes and droplet inhalation; in field laboratories, the staff were possibly bitten by mites (Table 2).

### Murine Typhus LAIs

There have been documented LAIs with murine typhus in 35 individuals contained in 10 reports from 1941 to 1995 (Tables 1 and 3). Interestingly, in contrast to scrub typhus LAIs, there were no reported deaths and the majority (97% [34/35]) were believed to be aerosol exposures with the exception of 1 self-inoculation incident. The reports detailed multiple infections mainly related to animal work and the aerosol exposures occurring during animal necropsy procedures. As with scrub typhus, there were no infections of people working near but outside the laboratories caused by laboratory incidents or procedural errors inside the laboratory.

**Table 3.** Summary of Published *Rickettsia typhi* Laboratory-acquired Infections

Year	Location	Route	No. Infected	Patient Details	Time to Illness Onset	Circumstances	Reference
1941	Switzerland	Aerosol	6	6 laboratory staff	NA	Producing murine typhus vaccine by harvesting and homogenizing rodent lungs.	[38]
1942	UK	Aerosol	12	12 laboratory staff	NA	Intranasal inoculation of mice with *Rickettsia typhi* (Wilmington strain) for experimental purposes during vaccine development.	[37]
1954	US	Aerosol	6	6 female ancillary staff	12 d	Autoclaves used to sterilize contaminated glassware were not operating satisfactorily despite gauges showing normal operation.	[42, 43]
1962	US	Aerosol	3	2 males, 1 female	9–14 d	Aerosol exposures in the laboratory and in hospital. Son of researcher infected.	[39]
1969–1970	US	Aerosol	3	1 male	NA	Not stated in detail.	[44]
1978	US	Suspected aerosol		1 female, 30 y		*Rickettsia typhi* experiment in biological safety cabinet, wearing gown, mask, and gloves. Vessels removed and placed on an open bench top for a period during the experiment, thereby exposing staff to *R. typhi* aerosols.	[45]
		Suspected aerosol		1 female, 32 y		Harvesting *R. typhi* from eggs, including purification, several steps of which involved the risk of aerosol generation. Mask only used intermittently.	
		Self-inoculation		1 male, 29 y		Accidental self-inoculation of finger with a needle used to inject steroids into mice infected with *R. typhi*.	
c. 1990	Korea	Suspected aerosol	1	1 female, 32 y	NA	Worked in a rickettsial disease laboratory performing *R. typhi* inoculation in mice and cell culture.	[46]
c. 1995	Malaysia	Aerosol	1	1 female, 34 y	4 d	Forced opening of Eppendorf tube caused accidental splashing of *R. typhi* onto her right eye and lips.	[47]

Abbreviations: NA, not available; UK, United Kingdom; US, United States.

In 1941, Van den Ende and colleagues [37], detailed a series of 4 murine typhus LAIs at the NIMR during efforts to produce a vaccine. They indicated that precautions were taken to prevent staff infections, such as the use of gauze masks during inoculation of mice, and mice being inoculated in a glass and metal box. It would also appear that there was some attempt to separate the areas where animal inoculations were performed from other areas of the laboratory. The report indicated that the most likely source of infection was “inhalation” during the intranasal inoculation of mice. They also noted that 1 staff member who became infected was not involved in the inoculation of mice, and they surmised that “dust-borne infection” may be a possibility [37].

Other activities that resulted in murine typhus infections were laboratory incidents, animal necropsy, and sample processing and equipment malfunction [33, 42, 43] causing aerosol exposure. LAIs with murine typhus were not restricted to laboratory staff; 1 incident resulted in the infection of a visitor [39]. This incident occurred in the laboratory of Dr Charles Wisseman at the Department of Microbiology at the University of Maryland School of Medicine, Baltimore, and was that of the 11-year-old son of one of the authors, who had accompanied his father into the laboratory on the weekend. The boy had briefly entered a restricted area of the laboratory where purified concentrated suspension of viable *R. typhi* had been prepared earlier in the day. Nine days later, the child demonstrated symptoms of murine typhus infection [39]. It is likely that this was due to the ubiquitous use of Waring blenders during this period, which produced significant aerosols in the processing of egg yolk sac material infected with *R. typhi*. Other incidents involving malfunctioning equipment were related to waste disposal via autoclaves where ancillary staff were exposed to *R. typhi* while washing Petri dishes that had been inadequately disinfected following an autoclave cycle [33, 42, 43].

Fourteen LAIs occurred from 1950 onward, and the last murine typhus LAI reported was in 1995 following the exposure of a laboratory technician to aerosol and infectious liquid [47].

## DISCUSSION

Scrub typhus and murine typhus have been associated with LAIs ever since the organisms have been the focus of laboratory investigations [22, 29, 48, 49]. However, the number of recorded cases of LAIs with these organisms are likely to be the tip of the iceberg, with the majority of the cases going unreported or undiagnosed.

Results presented here clearly demonstrate that the highest-risk activities for *O. tsutsugamushi* and *R. typhi* LAIs relate to working with infectious laboratory animals where significant aerosol exposures or bite-related infections may occur. The majority of these infections occurred prior to the 1950s when there were no antibiotic treatments available and the high infectious dose of the LAI incident likely contributed to the scrub typhus mortalities. In the case of aerosol-derived LAIs, these often occurred when staff were provided with only the most basic PPE such as gauze masks, which provided inadequate respiratory protection from infection [33, 37], or did not wear PPE. However, there has long been recognition of the risks associated with rickettsial disease research. In 1945, Buckland and Dudgeon [50] recommended primary and secondary containment biosafety practices to reduce the likelihood of *O. tsutsugamushi* LAI during the large-scale production of scrub typhus vaccine. In 1951, Smadel highlighted the hazardous nature of rickettsial research in the following points: (1) ignorant, careless, or indifferent worker; (2) poor laboratory practices; (3) poor architectural design of the laboratory or inadequate equipment; (4) certain pathogenic agents that are especially communicable in the laboratory (giving the example of Q fever); and (5) “unavoidable accidents” [24]. Smadel [24] further suggested control mechanisms to prevent LAIs and highlighted the important role of training and awareness on laboratory staff, stating that “perhaps one of the most important measures in preventing laboratory infections is the awakening of consciousness in the individual of dangers and to the realisation of the possibility that certain of these may be minimised by proper techniques.” In the 1950s and 1960s, the work of Sulkin and Pike [48] cataloging and determining the cause of LAIs led to the implementation of institutional biosafety programs and specific engineering solutions such as biological safety cabinets that have subsequently found their way into routine use in the majority of the laboratories worldwide.

From a biosafety and biocontainment viewpoint, all members of *Orientia* spp and *Rickettsia* spp are classified as risk group 3 (RG3) organisms and, as such, significant biosafety controls are placed on their manipulation, propagation, and storage. The classification of *Orientia* spp or *R. typhi* as RG3 organisms by the United States, the European Union, Australia, New Zealand, Belgium, Germany, and Switzerland [51] may be due to their association with the more pathogenic *R. rickettsii* and *R. prowazekii*—both of which were classified as US Select Agents in 2007 [52]; however, only the latter retains this status in the current list [53], likely due to its bioweapon potential [54]. While both *Orientia* spp or *R. typhi* can cause serious infections, it is difficult to maintain a strong argument for their classification as RG3 organisms, as they are treatable with antibiotics and do not spread in the community by person-to-person contact. To further compound biosafety restrictions on *Orientia* spp or *R. typhi* laboratory investigations, the mistaken belief that risk group is equal with biosafety level (BSL) [55] has resulted in the erroneous requirement of BSL-3 for *Orientia* spp or *R. typhi* work while not considering the risk associated with the individual procedures. BSL-3 containment laboratories fundamentally differ from BSL-2 laboratories in that they provide protection to the outside environment from accidental aerosol contamination but no additional user protection. BSL-3 laboratories provide protection to the outside environment by directional airflow via a cascading negative pressure gradient, and the exhaust air may or may not be high-efficiency particulate air filtered. BSL-3 containment laboratories are expensive to build and maintain due to their specialized nature and high energy requirements for heating, ventilation, and air conditioning systems in hot and cold environments.

It is likely that RG3 and BSL-3 restrictions have placed significant limitations on the progress toward scrub typhus and murine typhus research and the search for a vaccine. The risk-averse approach of management and regulators to the cultivation and propagation of *Orientia* spp and *R. typhi* and similar tropical infectious agents, even for low-risk activities, has resulted in the proliferation of high-containment laboratories in both relatively wealthy and developing countries. The need for high-containment laboratories for the majority of *Orientia* spp or *R. typhi* propagation has been overstated, and the mistaken requirement to perform all such work at BSL-3 laboratory facilities through rules imposed by national governments (should they exist), or foreign institutions providing donor funds, has further compounded the issue. These requirements place a strain on finances, laboratory infrastructure, and staff capacity on already stretched budgets, reducing investment in such research that is vital for improving diagnosis, treatment, and prevention of these neglected diseases. Academics fear being found in breach of national laws or incorrectly perceive the activities to be too “high risk” to be performed.

We believe there is a need for a risk-based approach to biological safety regarding the culture of *Orientia* spp and *R. typhi* and therefore suggest that the blanket risk classification of these organisms as RG3 coupled with BSL-3 containment requirements for all infectious activities should be revisited. At present, the most extensive guidance is provided by the US government [52], which states that BSL-2 practices, containment equipment, and facilities are recommended for nonpropagative laboratory procedures, and BSL-3 practices, containment equipment, and facilities are recommended for all other manipulations of known or potentially infectious materials. Recently, the World Health Organization (WHO) commissioned a revision of the current WHO Laboratory Biosafety Manual (LBM) using a risk-based approach for the fourth edition [55]. Using this approach, the LBM will suggest that only the most high-risk activities are required to be performed in laboratories with heightened control measures such as BSL-3 laboratory facilities. The scrub typhus and murine typhus LAI evidence presented here suggests that relatively low-risk activities, such as the inoculation of cell cultures or the early stages of in vitro growth with low volumes of infectious material at low concentration, can be performed within a biological safety cabinet located in core laboratories with BSL-2 containment and, when warranted, the requirement for RG3 PPE and practices should be maintained.

This is evidenced by the fact that scrub typhus and murine typhus LAIs have not been reported for nearly 20 years and that exposure of laboratory staff to infectious materials and their products are mitigated by strict personal biological safety control measures using administrative controls such as training and competency assessment as well as PPE. It is important to note that the reports presented in this review gave no evidence for scrub typhus or murine typhus infections in the community near but outside laboratories caused by laboratory incidents or procedural errors. This observation confirms that reasonably close contact with the infectious source is required to cause infection and that the need for BSL-3 laboratory containment is only required when performing high-risk procedures such as large-scale propagation or housing of infected animals.

Considering historical scrub typhus and murine typhus LAI infections, one has a great deal of respect for the courage of the researchers who were infected and, for those who subsequently died, it is particularly tragic. This was especially the case in the preantibiotic era where there was only rudimentary knowledge regarding the causes of such incidents with no treatment options available. It is our obligation to continue the research started by these brave researchers while bearing in mind the need for risk-based biosafety approaches and mitigation controls of the modern era.

## Notes

***Financial support.*** All authors are funded by the Wellcome Trust of the United Kingdom.

***Potential conflicts of interest.*** All authors: No potential conflicts of interest. All authors have submitted the ICMJE Form for Disclosure of Potential Conflicts of Interest. Conflicts that the editors consider relevant to the content of the manuscript have been disclosed.
